# Cost Comparisons between Home- and Clinic-Based Testing for Sexually Transmitted Diseases in High-Risk Young Women

**DOI:** 10.1155/2007/62467

**Published:** 2007-12-09

**Authors:** Kenneth J. Smith, Robert L. Cook, Roberta B. Ness

**Affiliations:** ^1^Section of Decision Sciences and Clinical Systems Modeling, University of Pittsburgh School of Medicine, Pittsburgh, PA 15213, USA; ^2^Departments of Epidemiology & Biostatistics and Medicine, College of Public Health and Health Professions, University of Florida, Gainesville, FL 32610, USA; ^3^Department of Epidemiology Graduate School of Public Health, University of Pittsburgh, Pittsburgh, PA 15261, USA

## Abstract

Home testing for chlamydia and gonorrhea increases screening rates, but the cost consequences of this intervention are unclear. We examined the cost differences between home-based and clinic-based testing and the cost-effectiveness of home testing based on the DAISY study, a randomized controlled trial. Direct and indirect costs were estimated for home and clinic testing, and cost-effectiveness was calculated as cost per additional test performed. In the clinic testing group, direct costs were 49/test and indirect costs (the costs of seeking or receiving care) were 62/test. Home testing cost was 25/test. We found that home testing was cost saving when all testing for all patients was considered. However cost savings were not seen when only asymptomatic tests or when patient subgroups were considered. A home testing program could be cost saving, depending on whether changes in clinic testing frequency occur when home testing is available.

## 1. INTRODUCTION

Home testing for chlamydia and gonorrhea has high sensitivity (about 90%) and specificity (>99%), and is well accepted in adolescent and young adult populations [1–3]. Studies in the US and in Denmark demonstrate that home testing significantly increases the likelihood that tests will occur [1, 2]. It is well established that office-based screening for chlamydia is a cost-effective intervention for sexually active young women [3–5], as is screening for gonorrhea in women with individual or population risk factors [6, 7], however screening in clinical settings is not performed as often as is recommended [8, 9].

Home screening, while increasing test frequency, might decrease screening costs, by avoiding clinical facility and clinician fees [1]. The indirect costs of clinic-based screening, such as time off work or school, childcare, transportation, and other costs resulting from a clinic visit, could also be averted. In this analysis, we use test frequency and cost data from a randomized trial of home testing for chlamydia and gonorrhea in high-risk young women to examine cost differences between home-based and clinic-based testing and the cost-effectiveness of a home testing intervention.

## 2. MATERIALS AND METHODS

The data source for this analysis was the Detection Acceptability Intervention for STD’s in Young women (DAISY) study, a randomized controlled trial. Trial recruitment and results are detailed elsewhere [1]. Briefly, 398 young women aged 15–24 at high risk for STD were recruited from clinics and surrounding communities, representing a group where frequent testing is recommended. Recruitment occurred between November 2000-April 2003. Women were randomized to an intervention group, who received home testing kits for chlamydia and gonorrhea by mail at 6, 12, and 18 months, or a control group, who received a postcard at 6, 12, and 18 months inviting them to attend one of the participating study clinics for a routine test for women’s health infections at no cost. Participants were urged to maintain their usual health patterns, including evaluation for genital symptoms or STD’s. Women with positive home tests were notified, counseled about partner notification, and referred to a participating clinic for treatment at no cost. Significantly more screening tests were performed in the home screening intervention group, and no differences in STD incidence rates were seen between intervention and control groups over the study period.

All participants completed a questionnaire at enrollment, which included questions about out-of-pocket costs for seeking or receiving care at the clinic, time off work or school due to clinic visits, and time donated by others to allow the participant to attend clinic. These questions and the number of tests performed in clinic and at home in the trial are the focus of this analysis.

The direct and indirect costs of receiving screening tests were calculated for women randomized to the intervention (testing at home) and for those in the control group (clinic testing). Direct costs of clinic-based testing were the costs for clinician time and for the test kit (nucleic acid amplification for chlamydia and gonorrhea), along with required supplies. The Panel on Cost-Effectiveness in Health and Medicine recommends including the following as indirect costs of care: payments made by the patient in the course of seeking or receiving care, and time required by the patient and others to allow care to be received [10]. In this analysis, indirect costs for the clinic visits included costs of parking or transportation and of babysitting or childcare. Indirect costs for time missed from school or work to receive care and for time donated by others (e.g., for rides or babysitting) to allow care to occur were quantified based on patient responses, then valued based on the average hourly wage in 2005 for nonfarm workers in the US [11] to avoid bias against intervention in this young population [10]; the effects of other time costs were examined in sensitivity analyses. Direct costs for home screening were the cost of the test, packaging costs, and postage to and from the patient, costing a total of 25. Testing cost alone (including materials and technician time) in either setting was 21. These costs were then applied to clinic-based and home-based tests as quantified in the DAISY study, and costs compared between intervention and control groups. Alternative values for all cost components, varied individually and collectively over plausible ranges, were examined in one-way and multiway sensitivity analyses.

Direct costs per subject for the intervention group and the control group were calculated as follows: Direct cost (clinic test) * clinic test frequency + Direct cost (Home test) * home test frequency divided by the number of subjects in each group. Indirect costs were calculated similarly.

Cost-effectiveness calculations were performed to estimate the cost per additional test performed per subject, using the formula

(1) $$\frac{\text{Total cost per subject (intervention)}-\text{Total cost per subject (control)}}{\text{Tests per subject (intervention)}-\text{Tests per subject (control)}}.$$

The model assumes equal STD incidence and diagnosis with home- and clinic-based testing, as seen in the DAISY trial, and hence the advantage of home testing from a cost-effectiveness standpoint (if any) is that, compared to the clinic testing group, more tests are performed overall due to testing at home (at a relatively low cost) while fewer tests are performed in clinic (at higher cost).

One-way and probabilistic sensitivity analyses were performed. Cost and time parameters were varied individually to examine effects on model results. In the probabilistic sensitivity analysis, these parameters were varied simultaneously, along with testing frequencies from the DAISY study, through their 95% confidence intervals, with values for each parameter randomly chosen from distributions 10,000 times and cost-effectiveness ratios calculated for each parameter set chosen. Parameter distributions were selected based on the characteristics of each parameter. Uniform distributions, where all values in the range were equally likely to be chosen, were used for time cost per hour, nonclinician direct costs for clinic-based testing, and home-based testing costs. Gamma distributions, based on DAISY study mean and standard deviation (SD) data, were used for indirect monetary costs, time required to receive care, and for clinician costs. Normal distributions were used for test frequencies.

## 3. RESULTS AND DISCUSSION

Questionnaire results are summarized in Table 1. Responses to these questions were similar regardless of randomization group, therefore results are reported for 388 subjects (10 of the original 398 subjects had no questionnaire data available). Slightly more than one-fifth of subjects had no insurance, took more than 2 hours off school or work to attend clinic, or traveled more that 30 minutes to attend clinic; more than a third paid for parking/transportation or had another person donate time so that care could be obtained.

Costs per test received are summarized in Table 2. Direct costs for clinic-based testing, including clinician and test costs, were estimated at 49 per test received. On average, subjects paid 2.97 for parking/transportation and childcare, and 3.7 hours were spent by the subject and others to allow care to be received per clinic visit. Combined direct and indirect costs per clinic visit totaled 111.

Table 3 displays tests performed, testing costs, and cost-effectiveness results for all study subjects and for subgroups based on recruitment site. More tests and more asymptomatic tests were performed among the home screening group (P<.0001), with greater differences in testing rates seen in women recruited from neighborhoods. Women recruited from clinics had high testing rates and a smaller differential between home and clinic-based testing. No difference in STD incidence was noted between intervention groups (20.4 [home testing] versus 24.1 [clinic testing] per 100 woman-years, P=.28).

Considering total costs for all subjects and all testing, and assuming equal STD detection between groups, the home testing intervention was cost saving, because fewer clinic-based tests were performed and the resulting cost savings were not completely offset by the costs of increased home testing (Table 3). Total testing costs per infection found were estimated at 702 in the intervention group and 717 in the control group. However, cost savings were not noted for the neighborhood recruitment subgroup, where each additional test obtained by the intervention group compared to the control group cost about 24.50, due to more frequent home-based testing and no decrease in clinic testing with the intervention. When only asymptomatic testing was considered in all subjects, the intervention cost was 12.51 per additional test performed, since the costs of increased home testing were not offset by the relativity small decreases in clinic-based testing in the intervention group.

In one-way sensitivity analyses, four parameter values varied individually through their listed ranges (Table 2) made the total cost per subject of all testing in the intervention group greater than the total cost per subject in the control group (Table 4). The model was most sensitive to changes in home testing costs, with increases in this cost of ≥5 making the home testing intervention more expensive than clinic testing; to have similar effects, time spent seeking/receiving care, the cost of that time, or clinic testing cost would need to decrease by about a third. Most importantly, if infection detection frequency is not equal between groups, unlike the equal detection seen in the DAISY trial and assumed in this analysis, between-group differences in infection costs and complication effects would need to be explicitly accounted for in the analysis.

In the probabilistic sensitivity analysis, where all cost and testing parameters were varied simultaneously, the intervention was cost saving in 52% of model iterations when all testing was considered. When considering only asymptomatic testing, there was a 22% likelihood that the intervention would be cost saving.

**Figure 1:**
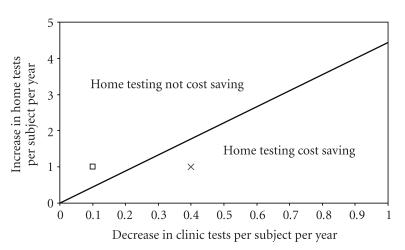
Two-way sensitivity analysis on changes in testing rates resulting from a home testing intervention. The line represents points where the overall costs of a home testing program or clinic-based testing are equal when gonorrhea and chlamydia detection rates are the same with either program. Points denoting changes in home and clinic testing frequency occurring due to a home testing program that fall in the area below the line (e.g., the “X”) indicate that cost savings would occur with that program. Points above the line (e.g., the open square) denote parameter values where cost savings would not occur.

Figure 1 illustrates the relationship of changes in testing rates to cost savings with a home testing program if infection detection rates are equal between groups. If per-patient clinic testing rates decrease by more than 22.5% of the increase seen in home testing rates due to program adoption, a home testing program will be cost saving. For example, if a home testing intervention increases home testing in a population by 1 test per patient per year and decreases yearly clinic testing by 0.4 tests per patient (denoted by the “X” in Figure 1), then the intervention would be cost saving. However, with the same increase in home testing, if the clinic testing rate only decreases by 0.1 test per patient (denoted by the open square), then a home testing program is not cost saving.

In this analysis of a program encouraging home testing for chlamydia and gonorrhea in a high-risk group of young women, we found that, when all costs and all tests were considered, a home testing program was cost saving in the DAISY study, a randomized, controlled trial, while, at the same time, significant increases in all tests and in asymptomatic tests were seen. However, whether cost savings occurred with home testing depended on the patient group and clinical situation studied, based on changes in clinic-based testing resulting from the intervention when STD detection is the same with either testing program. Cost savings were seen in clinic-recruited subjects, a group that utilized clinic services more frequently, due to decreases in clinic-based testing resulting from the availability of home testing. In subjects recruited from neighborhoods surrounding the clinics, home testing was not cost saving, because of increased home testing without proportionate clinic testing reductions or STD detection improvements. When only asymptomatic tests were considered, home testing was again not cost saving. Finally, the indirect costs of seeking or receiving care, that is, monetary costs for childcare, parking, and other expenses; time costs from missed work or school; and time donated by others were considerable and could present barriers to receiving recommended testing and care.

As suggested by this analysis and by the DAISY study itself, the decision to implement a home testing intervention for chlamydia and gonorrhea is more complex than merely seeking to maximize tests performed or to minimize costs. Home testing programs have been well demonstrated to increase testing volume [1–3], by decreasing some barriers to STD testing through the use of a relatively expensive test. Unless direct costs incurred by individual clinics could be decreased as a result of a home testing intervention, in times of budgetary limitations clinics would have no incentive or means to implement such an intervention. Indirect cost savings (when the intervention is considered from the broader, societal standpoint) and the public health benefits of increased testing suggest that financial support for home testing would need to come from higher levels for these societal benefits to be obtained. For individual clinics with a high proportion of frequently utilizing patients, a home testing intervention could decrease the visit frequency by this patient group, increasing capacity for other needed services and patient groups. In different populations where care-related indirect costs might be higher, for example, due to clinic inaccessibility or unavailability, home testing might prove more useful from clinical and economic standpoints by increasing testing rates through decreasing individual disincentives for testing. Concerns about home testing decreasing necessary clinic visits appear to be unfounded [1].

Cost savings were not seen when only asymptomatic tests are considered, implying that home testing for screening purposes may not be economically favorable. However, the distinction of symptomatic and asymptomatic testing might be somewhat artificial when a population is considered, since the availability of home tests could have benefits beyond those of screening. For example, delays in seeking care when symptomatic are common, and the availability of home testing may allow infections to be diagnosed sooner, potentially decreasing untreated illness burden and PID risk. No differences in infection detection were seen between randomization groups in the DAISY study, where an urban population with relatively easy and frequent access to care was investigated; populations with less access could benefit more from home screening. Finally, few screening interventions are cost saving, with some cost per health benefit gained absorbed by society or payers based on the magnitude of screening costs and benefits gained [12]. For example, Hu et al. [4] found that annual chlamydia screening cost 2350 per quality adjusted life year gained compared to no screening and thus would be considered very cost-effective compared to other health care interventions. In our analysis, where equal infection rates are seen between intervention groups, the cost per health benefit gained depends on the benefits of greater testing frequency for infected women and their partners. Unfortunately, these benefits cannot be estimated based on present data, a limitation of our analysis.

## 4. CONCLUSIONS

A home testing intervention for chlamydia and gonorrhea, while increasing testing rates, has the potential to be cost saving when the direct and indirect costs of avoided clinic visits are considered. The cost equation depends in large part on whether changes in clinic testing frequency and STD detection occur as a result. Home testing effects on clinic testing frequency in other populations and on individual and population health in localities with more limited health services require further research.

## Figures and Tables

**Table 1:** Demographic and economic questionnaire data from the DAISY study.

	Frequency	Percent
All subjects	388	100%
Average age (years)	18.9	—
*14–18 years old*	*181*	*46.6%*
*19–25 years old*	*207*	*53.4%*
Recruitment site		
*Clinic*	*198*	*51.0%*
*Neighborhood*	*190*	*49.0%*
No insurance	86	22.2%
Paid for parking/transportation	136	35.1%
*Paid 5 or more*	*49*	*12.6%*
Paid a babysitter	25	6.4%
Other person donated time	143	36.9%
*Donated* ≥1 *hours*	*115*	*29.6%*
Took time off from work or school	136	35.1%
≥2 *hours*	*86*	*22.2%*
Clinic visit took ≥2 hours	99	25.5%
Travel time to clinic >30 minutes	86	22.2%

**Table 2:** Direct and indirect costs per screening test received and ranges examined in sensitivity analyses.

	Base case	Range
*Costs of clinic screening*		
Direct costs		
Clinician cost	28	12–50
Test cost	21	10–32
Indirect costs		
Monetary (mean [SD])*	2.97 [7.55]	0–25
Time (hours) (mean [SD])^†^	3.7 [2.7]	0.4–10.5
Time value (per hour)	16	7–25
*Costs of home screening*		
Direct costs		
Test cost, packaging, and postage	25	15–35

* Parking/transportation plus babysitter/childcare.

^†^ Time off work or school, plus time donated by others so that care could be received.

**Table 3:** Number of tests completed, testing costs, and cost-effectiveness of the home screening intervention.

		Tests completed				Cost per subject			Cost per additional test completed
		Clinic	Home	Total	Per subject	Direct	Indirect	Total	

*All tests*									
All subjects	Clinic testing (n=191)	511	2	**513**	2.7	132	166	**298**	—
	Home testing (n=197)	460	254	**714**	3.6	147	145	**292**	Cost saving

Clinic recruits	Clinic testing (n=99)	395	1	**396**	4.0	197	248	**445**	—
	Home testing (n=99)	337	119	**456**	4.6	198	212	**409**	Cost saving

Neighborhood recruits	Clinic testing (n=92)	116	1	**117**	1.3	62	78	**141**	—
	Home testing (n=98)	123	135	**258**	2.6	96	78	**174**	24.50

*Asymptomatic tests*									
All subjects	Clinic testing (n=191)	274	0	**274**	1.4	71	89	**160**	—
	Home testing (n=197)	261	173	**434**	2.2	87	82	**169**	12.51

Clinic recruits	Clinic testing (n=99)	199	0	**199**	2.0	99	125	**224**	—
	Home testing (n=99)	183	77	**260**	2.6	110	115	**225**	2.16

Neighborhood recruits	Clinic testing (n=92)	75	0	**75**	0.8	40	51	**91**	—
	Home testing (n=98)	78	96	**174**	1.8	64	49	**113**	23.13

**Table 4:** One-way sensitivity analysis, all subjects and all testing. Parameter values where the home testing intervention is more costly than clinic-based testing.

Parameter	Baseline value	Home testing more costly
Time spent receiving care		
Hours	3.7	<2.5
Cost per hour	16	<11
Testing costs		
Home	25	>30
Clinic direct costs (clinician & test costs)	49	<32
